# Radiological features of double and inverted mesiodens: a systematic review of the literature and presentation of two cases

**DOI:** 10.4317/medoral.27513

**Published:** 2025-10-14

**Authors:** Antonio Lo-Casto, Vera Panzarella, Ettore Palizzolo, Rodolfo Mauceri, Giovanna Giuliana, Francesco Bencivinni, Gaetano La-Mantia, Giuseppina Campisi, Manfredi De-Angelis, Giuseppe La-Tona, Olga Di-Fede

**Affiliations:** 1 Department of Biomedicine, Neurosciences and Advanced Diagnostics (BI.N.D.), University of Palermo, Italy; 2 Department of Precision Medicine in Medical, Surgical and Critical Care (Me.Pre.C.C.), Palermo, Italy; 3 Unit of Oral Medicine and Dentistry for frail patients, Department of Rehabilitation, fragility, and continuity of care, Regional Center for Research and Care of MRONJ, University Hospital Palermo, Palermo, Italy; 4 Dental and Maxillofacial Radiology (U.O. 69.01.4), A.O.U.P. "P. Giaccone" of Palermo, Italy; 5 Department of Biomedical and Dental Sciences and Morphofunctional Imaging, University of Messina, Messina, Italy

## Abstract

**Background:**

Mesiodens are the most prevalent supernumerary teeth, defined as a dentition anomaly. Mesiodens account for 80% of all supernumerary teeth, with a prevalence in the general population of 0.15% -1.9%. Mesiodens show a higher frequency in man than in women, it is usually found in the front upper jaw, between the midlines of maxillary central teeth. It may be normally oriented or have inverted and transverse orientation. Single or multiple mesiodens may occur; however, double mesiodens is rarer (10-19% of all patients with mesiodens), and even rarer is its double inversion, with the crown and root oriented towards the nasal and oral cavities. The aim of this study is to investigate the main cone-beam computed tomography (CBCT) radiological features of patients affected by mesiodens. Two cases of double mesiodens, one of which with double inversion, in pediatric patients are reported.

**Material and Methods:**

According to PRISMA guidelines, a systematic review search was conducted on PubMed, Scopus, and Web of Science. Observational studies conducted on patients with double mesiodens were selected and analyzed, including both radiological features and clinical characteristics. Furthermore, two cases of patients with double mesiodens were reported.

**Results:**

Fourteen studies were included, and 14 patients affected by double mesiodens were analyzed (12 males, 2 females, with a mean age of 13.5 ± 6.4 years).

**Conclusions:**

The present study underscores the importance of considering the CBCT a fundamental investigation to define and appropriate diagnosis and management, and to avoid complications related to a late diagnosis and/or an unnecessary surgical approach.

** Key words:**Mesiodens, tooth abnormalities,cone beam computed tomography, CBCT, diagnosis.

## Introduction

Among the tooth abnormalities, supernumerary tooth is a dental element with an atypical morphology and anomalous location, and it is not therefore attribuTable to any dental element of the physiological series [1,2].

Supernumerary teeth may be found anywhere in the mouth; however, they mainly involve the upper jaw. They may occur as a single tooth or multiple teeth, unilaterally or bilaterally, erupted or impacted and in mandible/maxilla or both the jaws. Multiple supernumerary teeth are also associated to other diseases or syndromes [1,2].

Supernumerary teeth maybe present in primary and permanent dentitions, with a prevalence of 0.8% and 2.1%, respectively[3]. However, some studies reported a prevalence over 5% in the permanent dentition among Caucasians [4,5].

Supernumerary teeth may be classified considering several features. Chronologically, they can be classified as pre-deciduous, like permanent teeth, and post permanent or complementary; morphologically as conical, tuberculate, infundibuliform, supplemental (eumorphic) and odontome; topographically as mesiodens, paramolar, distomolar and parapremolar, and according to orientation as vertical, inverted and transverse [1,6].

Among supernumerary teeth, mesiodens account for 80% of all supernumerary teeth, with a prevalence in the general population of 0.15% -1.9%. Mesiodens show a higher frequency in man than in women (2:1 ratio)[5,7-9].

It is usually found in the front upper jaw, between the midlines of maxillary central teeth. It may be normally oriented or have inverted and transverse orientation. Single or multiple mesiodens may occur [5,7-9].

**Figure 1:**
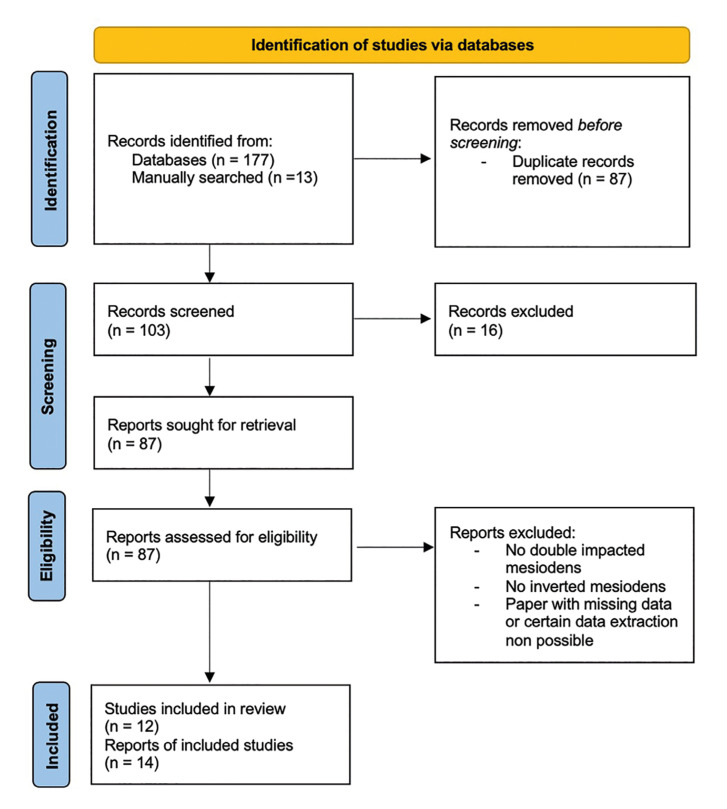
Flowchart of the different phases of the systematic review.

**Figure 2:**
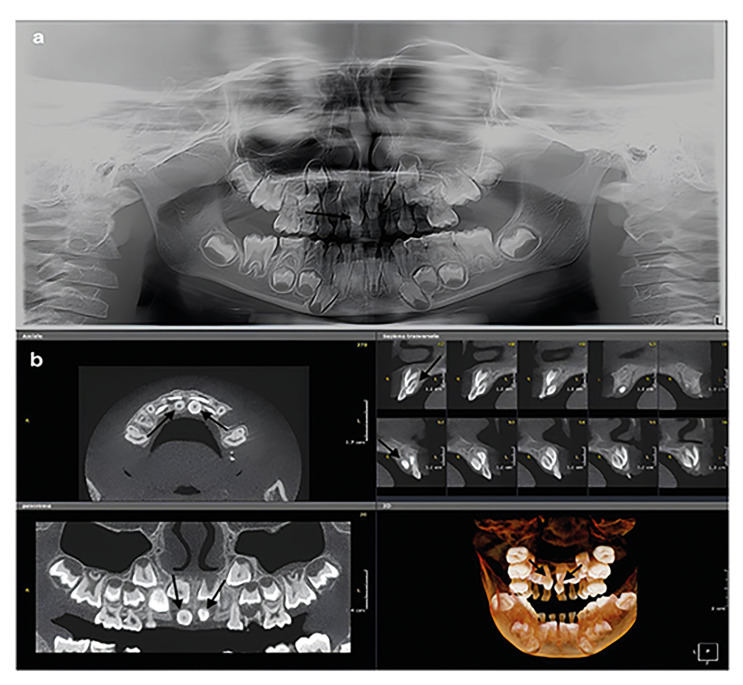
Double mesiodens in a 5-year-old girl. (a) Panoramic radiograph. Two supernumerary teeth (arrows) are appreciable between 11 and 21 above, and 51 and 61 below. (b) CBCT. The two mesiodens (arrows) are vertical, palatal oriented, had a conoid shape and are contiguous with the nasopalatine canal.

Double mesiodens is rarer (10-19% of all patients with mesiodens), and even rarer is its double inversion, with the crown and root oriented towards the nasal and oral cavities (Fig. 1, Fig. 2) [10].

Mesiodens is usually asymptomatic and included, commonly discovered during routine radiological exams [5,9]. Mesiodens may be associated to several oral conditions, they can lead to crowding, delayed or prevented eruption of central incisors, midline diastema, root resorption, axial rotation and cyst formation [5].

Panoramic and dental x-rays may be helpful for diagnosis, orthodontic and surgical management of mesiodens. However, conventional dental radiographic exams do not provide the accurate information about the three-dimensional position of mesiodens [11]. Cone-beam computed tomography (CBCT) delivers the exact position of mesiodens and its contact with the other teeth and contiguous anatomic structures (e.g. nasal cavity, nasopalatine canal). The latter could be fundamental to define the treatments strategies and, if dental surgery is indicated, it may help oral surgeon in the choice of the surgical access [5].

The present systematic review investigates the main radiological findings in patients with mesiodens, and to define the appropriate diagnosis of this unusual conditions performed. Moreover, two new cases of double mesiodens, in pediatric patients, of which one with both mesiodens inverted are reported.

## Material and Methods

- Protocol

A systematic literature search was conducted independently by two of this paper’s authors (RM and EP). The protocol for this study was designed following the Preferred Reporting Items for Systematic Reviews and Meta‐Analyses (PRISMA) guidelines (PROSPERO ID: 626903) [9].

- SPIDER and research question

The research question was designed based on SPIDER item, consisting of the following information:

Sample (S): Patients diagnosed with double and/or inverted mesiodens.

Phenomenon of Interest (PI): Radiological characteristics used to support diagnosis.

Design (D): Observational studies, including case reports and case series.

Evaluation (E): Description and assessment of radiographic features relevant to diagnosis.

Research type (R): Systematic review.

The systematic review was based on the following research question: "What are the radiological characteristics of double and inverted mesiodens of general population?"

- Data sources and search strategy

The selection of studies concerning patients affected by double and inverted mesiodens with a radiologically confirmed diagnosis was performed.

Records were identified using different search engines (e.g., Medline/PubMed, Scopus, and Web of Science) and by scanning reference lists of articles, covering the period from 2000 to 2024.

For the search strategy, MeSH terms and free-text words were combined through Boolean operators as follows: “Tooth, Abnormalities” (MeSH), “mesiodens”, “inverted”,”Radiograph”, “Dental imaging”, "Cone-Beam Computed Tomography".

The research was completed in April 2024.

- Eligibility criteria

The inclusion criteria for the studies were as follows:

1. Human studies;

2. English language;

3. Studies including patients with at least two included mesiodens in the maxillary anterior region;

4. At least one inverted included mesiodens (crown direction);

5. Radiological investigation by conventional radiography and/or CT/CBCT.

These criteria were selected to ensure a focused and standardized approach to data inclusion was followed. Human studies were prioritized to maintain clinical relevance, and the requirement for research presented in the English language ensured consistency in data interpretation.

The strict inclusion of radiological investigation was intended to enhance the precision and reliability of the study outcomes. In establishing these criteria, careful consideration was given to ensure the selection of studies that could provide robust and reliable data relevant to the objectives of the study and to ensure their reproducibility.

The exclusion criteria were: (I) less than two mesiodens in the maxillary anterior region; (II) research involving animals and *in vitro* studies; (III) systematic and narrative reviews, expert opinions; and (IV) studies written in languages other than English. Research results not satisfying the inclusion criteria were excluded during data collection. Risk of bias was evaluated for all included studies (Supplement 1).

- Study selection and data collection processes

The initial search identified 177 records, additionally 13 papers were manually searched. From the 190 papers recognized, 87 were removed as they were duplicates. The screening of 103 studies was performed based on the titles and abstracts, and 16 records were excluded. Subsequently, a full‐text evaluation of 87 studies was carried out. Finally, based on the inclusion criteria, 75 records were excluded, and 12 papers were included in the current review; a detailed flow chart of the selection process is provided in Fig. 1.

The eligibility assessment was performed independently by two reviewers (R.M. and E.P.) and any discrepancies between reviewers were resolved by consensus. The studies were initially selected by applying the inclusion and exclusion criteria of the study and Abstract titles. Duplicate papers were deleted, after which there followed further scrutiny to assess their eligibility.

Regarding the outcomes and all other variables retrieved from the included papers, we collected: “reference” (i.e., author, year, country), “patient” (i.e., sex, age) and “mesiodens findings” (i.e., imaging technique, eruption status, position, number of inverted mesiodens, shape and associated abnormalities). Any missing data were described as “not defined (n.d.)”.

- Case reports

The study protocol conformed to the ethical guidelines of the 1964 Declaration of Helsinki and its later amendments or comparable ethical standards. It was also approved by the Institutional Local Ethics Committee of the University Hospital “P. Giaccone” in Palermo (Palermo, Italy) (approval #3/2013). All patients signed written informed consent.

- Data collection and clinical and radiological examinations

The clinical procedures were performed at the Dentistry Unit of the University Hospital “Paolo Giaccone” in Palermo (Italy).

During the interview, variables including sociodemographic data and medical history were recorded.

Radiological investigations (i.e., panoramic radiograph and CBCT) were performed at the Unit of Dental and Maxillofacial Radiology of the University Hospital “P. Giaccone” in Palermo (Italy).

## Results

- Systematic review

Of the 87 total records assessed for eligibility, only 12 were selected since they satisfied the inclusion criteria. The main characteristics of the included studies are reported in Table 1.

All the included articles were observational studies published between 2007 and 2023. Of the 13 studies, 8 were from India [10,12-17], 1 was from Turkey [18], 1 was from Tunisia [19], 1 was from Saudi Arabia [20], 1 was from Iran [21]and 1 was from Japan [22].

In total, 14 patients affected by double mesiodens were analyzed, of which 12 were males and 2 were females. Based on the available data, the mean age of patients with double mesiodens was 13.5 ± 6.4 years. The age of patients affected by mesiodens ranged from 7 to 32 years.

Regarding the radiological exam, most of the authors investigated the mesiodens at least by mean of 2 different techniques (12/14, 85.7%). Panoramic radiograph was the most used exam (10/14, 71.4%), followed by maxillary occlusal radiograph (6/14, 42.8%), CBCT (5/14, 35.7%), CT (4/14, 28%), periapical x-ray (3/14, 21.4%) and lateral cephalogram (2/14, 14,2%).

Regarding the orientation of the double mesiodens, half of the patients had both mesiodens inverted. Concerning the associated abnormalities highlighted by the Authors, most of the mesiodens were associated to a dentigerous cyst (5/14, 35.7%); the other more frequent abnormalities described in the included papers were: 5/14 (35.7%) abnormalities of tooth position (e.g., malocclusion, crowding, rotation); 6/14 (42.8%) problem of the permanent eruption (e.g., impacted canine, delayed eruption); and 3/14 (21.4%) swelling (Table 1).

- Case reports

**Figure 3:**
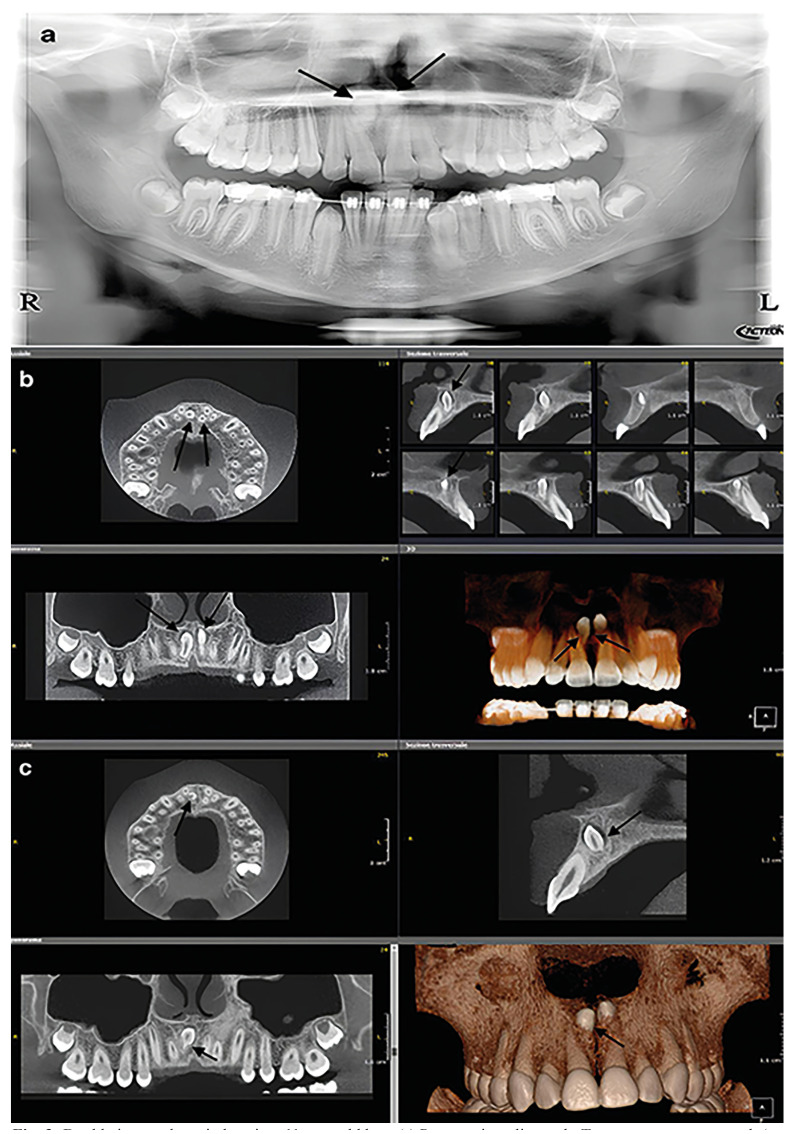
Double inverted mesiodens in a 11-year-old boy. (a) Panoramic radiograph. Two supernumerary teeth (arrows), partially overimposed to the hard palate, are seen between 11 and 21, whose roots are obliquely angled and distally displaced. (b) CBCT. The conoid shape of the two inverted mesiodens (arrows) is appreciable. Moreover, the palatal position, the relation with the nasopalatine canal and the contiguity of the 21 crown with the above nasal floor are demonstrated. (c) CBCT, 1-year follow-up. The right inverted mesiodens shows mild root resorption (arrow) compared to the initial CBCT (R=right; L=left).

The main findings in the two patients with double and inverted mesiodens are summarized in Table 2. The radiological exams of the patients included in this study are reported in Fig. 2 and Fig. 3.

Case report 1

A 5-year-old female was referred for orthodontic evaluation. On panoramic radiograph (OP 3D Pro, KaVo, Instrumentarium Dental, Palodex Group OY, Tuusula, Finland) two supernumerary teeth were appreciable between 11-21 and 51-61.

A CBCT (OPD 3D Vision, Kavo, Palodex Group, OY, Tuusula, Finland) was achieved to assess the relation with contiguous teeth and surrounding anatomic structures. The two impacted mesiodens were vertical, palatal oriented, with a conoid shape (Fig. 2).

Case report 2

An 11-year-old boy was orthodontically evaluated with the same radiological techniques of the previous patient. On panoramic radiograph, two supernumerary teeth were visible between 11-21. A CBCT revealed a double inverted, impacted mesiodens. Oblique CBCT reformations showed one mesiodens attached to the buccal cortical plate; the other one laid below the floor of the nasal cavity (Fig. 3).

## Discussion

A supernumerary tooth is defined as a dental element that has an atypical morphology, an anomalous location and is not therefore attribuTable to any dental element of the physiological series. Morphologically, a supernumerary tooth is distinguished according to its shape into conoid, tuberculate, infundibuliform, supplemental (eumorphic) and odontome. However, first three shapes are more commonly find. A conoid tooth is small with a cone-shaped crown and a squat root, often curved A tuberculated tooth is characterized by a crown with a variable number and variously oriented cusps or tubercles. An “infundibuliform” tooth has a crown with a typical introflexion which gives a funnel-shaped appearance and a single, conical root [1,6].

Supernumerary tooth, also defined as hyperdontia, in permanent dentition is more frequent in the incisive region, followed, in descending order, by the molar, premolar and canine region, in the upper maxilla, and premolar region, with a frequency slightly lower than the incisive, molar and canine region, in the mandible [1,6].

Mesiodens is sometimes associated to deciduous or permanent teeth retention, eruptive delay, ectopic eruption, dental malposition (rotation, inclination), and possible evolution in a dysplastic or cystic condition [20].

Several studies from different parts of the worldwide have shown a higher incidence in male than women, with male enrolment ranging from 64% to 75.2% [23-28].

Regarding the study included in the present review and our case reports, there is an even higher proportion of male (14/18, 77.8%).

Usually, mesiodens is diagnosed in pediatric patients, between the 6th and the 8th years of life [23-26]. However, there are also large observational studies with wide age ranges in enrolled patients, ranging from 3 to 58 years [5,28].

The latter is consistent with the data of reviewed literature (mean age of patients is 13.5 years, if we consider also our two cases is 12.9 years), with a range from 7 to 32 years.

According to the number, single mesiodens is reported in most of the cases, double mesiodens is rare, the occurrence of three mesiodens in a non-syndromic patient is very rare [5,24,25,28].

Regarding the orientation, inverted mesiodens ranges between 11% and 39% in larger series [5,23,25,27]; interestingly, in East Asia several studies have shown incidence rates above 50% of the enrolled patients, and up to 80% [24,28].

Among mesiodens pathogenesis, different theories have been proposed. Atavism (phylogenetic reversion) relies on the fact that the ancient relic ancestors had three central incisors. Dichotomy, refers to a tooth bud, split into two separate teeth, occurring from complete gemination in the anterior maxilla region. Palatal offshoots or hyperreactivity of active dental lamina are induced to develop into an extra tooth bud, which results in a supernumerary tooth developing into another extra supernumerary tooth. Because mesiodens have been diagnosed in siblings, twins, and in a single family, also genetics should have a role. Autosomal dominant inheritance with sex-linked patterns with incomplete penetration has been proposed [10].

Double inverted mesiodens is also an extremely rare event, only 8 times have been reported in the paper included in the present review of literature (Table 1).

A comprehensive evaluation of radiological findings across the included studies reveals distinct diagnostic contributions of the various imaging modalities employed. Conventional radiographs—such as panoramic, periapical, and occlusal views—are often used as first-line tools due to their accessibility, rapid acquisition, and relatively low radiation exposure [10,20].

These techniques allow an initial identification of supernumerary teeth and provide basic information regarding the presence, number, and general orientation of mesiodens. However, their two-dimensional nature imposes significant limitations, particularly in cases of impacted, inverted, or multiple mesiodens. In such scenarios, overlapping structures may obscure key details, and spatial relationships with adjacent anatomical landmarks may be inaccurately represented [11].

By contrast, cone-beam computed tomography (CBCT) offers a three-dimensional reconstruction of the maxillofacial anatomy, enabling clinicians to precisely determine the exact position, orientation, and depth of inclusion of the mesiodens. This is especially critical in double and/or inverted cases, where conventional imaging may fail to reveal relationships with nearby anatomical structures such as the nasopalatine canal, nasal floor, or adjacent roots.

In the included studies, CBCT was particularly useful in identifying complications such as root resorption, dentigerous cysts, and deviation or impaction of the permanent central incisors—findings that have direct implications for clinical management.

Most of the included study investigated the mesiodens at least by mean of 2 different techniques, and panoramic radiograph was more often performed, and computed tomography, either CT or CBCT, was performed in about half of the cases, and CBCT was the preferred technique 31.3% of cases [10,20].

CBCT allows a dose reduction with respect to helical CT, and this is especially important in pediatric patients. Additionally, and most important, CBCT provides a clear 3D detail of anatomical structures; the chance to define the exact number and position of mesiodens is fundamental [5,18].

From a treatment planning perspective, the radiological modality selected directly influences the decision-making process. CBCT not only enhances diagnostic accuracy but also assists in surgical planning by allowing virtual assessment of the most suiTable surgical approach (palatal, buccal, or transnasal), minimizing the risk of iatrogenic injury. Moreover, in asymptomatic cases or those not requiring immediate intervention, CBCT enables precise monitoring of the lesion over time, offering a reliable method for evaluating potential changes in position, morphology, or pathological transformation.

Treatment strategy is usually based on multidisciplinary management (e.g., oral surgeon and orthodontist), often the mesiodens need to be removed to correct any abnormalities of tooth position and/or problem of the permanent eruption, that may result in aesthetic problems [22]. In this scenario, the CBCT is useful for surgeon to define the surgical access to the lesion. Mesiodens is commonly approached orally with palatal flap, buccal flap, or both [13,15,16,18,19].

An intraoral transnasal approach is preferred when mesiodens erupts into the nasal cavity [21]. Also, a 3D image can be obtained from CT data acquired preoperatively and projected as a hologram to achieve mixed reality before and during surgery [22].

In case of unerupted permanent tooth, the 3D evaluation is extremely important also to correctly define the orthodontic treatment and to prevent any possible related complication (e.g., root resorption).

However, surgical therapy is not the mandatory treatment for impacted mesiodens, especially if it does not cause significant symptoms or complications to adjacent dental or other anatomic structures.

In our cases, no treatment has been managed for the absence of symptoms and other related conditions. According to the parents, it has been decided for the double inverted mesiodens case to treat only dental-alveolar discrepancy of lower arch and a rapid palatal expansion to increase the upper arch's transverse dimension thanks to a bearable misalignment. In the other case, no treatment has been planned for parent’s choice.

This study has some limitations derived from the small number of included patients, which was probably due to the rarity of the pathology investigated. Although double inverted mesiodens is not a common condition, our study emphasizes the importance of a CBCT investigation to identify all the radiological features of them, that are essential for enhancing early diagnosis, appropriate treatment.

## Conclusions

Double and/or inverted mesiodens must be correctly detected to perform appropriate diagnosis and management, and to avoid complications related to a late diagnosis and/or an unnecessary surgical approach.

Understanding the radiological characteristics of double and/or inverted mesiodens is essential for establishing an appropriate multidisciplinary management plan.

CBCT plays a pivotal role in accurately assessing their three-dimensional localization and supporting precise treatment planning, particularly in complex cases such as inverted or deeply impacted mesiodens.

## Figures and Tables

**Table 1:** The characteristics of the studies included in the literature review.

Reference			Patient		Mesiodens findings					
Author	Year	Country	Sex	Age (y)	Imaging technique	Eruption status	Position	N. inverted mesiodens	Shape	Associated abnormalities
Dinkar et al. (12)	2007	India	F	14	Maxillary occlusal radiograph Panoramic radiograph	Impacted	Maxillary anterior region between two permanent incisors	2/2	Both conical	Painfull swelling. Dentigerous cyst 4.5x4 cm
Canoglu et al. (18)	2009	Turkey	M	8	Panoramic radiograph Lateral cephalogram	Impacted	Maxillary anterior region between and buccally the two permanent incisors	2/2	n.d.	Crowding in the maxillary anterior region with axial rotation of permanent incisors
Sharma et al. (13)	2010	India	M	12	Maxillary occlusal radiograph Periapical radiographs	Impacted	Maxillary anterior region, one in close proximity to the periapical area of the immature root of left central incisor and the other was in relation to the impacted right central incisor	1/2	n.d.	Immature root of left central incisor Dentigerous cyst, approximately 10 mm diameter
Gharote et al. (14)	2011	India	M	10	Maxillary occlusal radiograph	Impacted	Maxillary anterior region	1/2	One conical, one tuberculate	Radicular cyst
			M	20	Maxillary occlusal radiograph	Impacted	Maxillary anterior region	1/2	Both conical	n.d.
Agrawal et al. (15)	2012	India	M	11	CT	Impacted	Maxillary anterior region, left side	1/2	n.d.	Dentigerous cyst
Byatnal et al. (16)	2013	India	M	13	CT	Impacted	Maxillary anterior region	2/2	n.d.	Asymptomatic swelling in the upper anterior jaw. Dentigerous cyst with buccolingual destruction of cortical plates (3 cm x 4 cm).
Mohan et al. (17)	2013	India	M	32	Maxillary occlusal radiograph Panoramic radiograph	Impacted	Maxillary anterior region, right side	1/2	n.d.	Trauma with teeth loss 10 yrs before examination. Palatal swelling. Dentigerous cyst Impacted right maxillary permanent canine 13
Omami et al. (19)	2015	Tunisia	F	8	Panoramic radiograph CBCT	Impacted	supernumerary tooth was in palatal position over the impacted permanent maxillary central incisor	1/2	n.d.	Delayed eruption of her right permanent maxillary central incisor (absence of the right maxillary permanent central and lateral incisors) Previous removal of a supernumerary tooth delaying the eruption of the left permanent maxillary central incisor (1yrs before)
Al-Sehaibany et al. (20)	2016	Saudi Arabia	M	8,5	Panoramic radiograph CBCT	Impacted	Maxillary anterior region between two permanent incisors	2/2	Both conical	Asymptomatic. Delayed eruption of 21. Perforation of the nasal fossa floor
Sharifi et al. (21)	2021	Iran	M	9	Panoramic radiograph CBCT	Impacted	Anterior region of the nasal floor and close to the anterior nasal spine (ANS) and the piriform aperturein Proximity to the roots of the adjacent permanent incisors.	2/2	n.d.	n.d.
Rajaram Mohan et al. (10)	2022	India	M	21	Panoramic radiograph CBCT	Impacted	Maxillary anterior region between two permanent incisors	2/2	n.d.	Asymptomatic. Missing upper front teeth. Extension into the floor of the nasal cavity
Koyama et al. (22)	2023	Japan	M	9	Panoramic radiograph CT	Impacted	Palatal side	2/2	n.d.	Maloclussion
			M	7	Panoramic radiograph CT	Impacted	Labial side	1/2	n.d.	n.d.

**Table 2:** Main findings of patients reported in the present study.

Patient		Mesiodens findings					
Sex	Age (y)	Imaging technique	Eruption status	Position	N. inverted mesiodens	Shape	Associated abnormalities
F	5	Panoramic radiograph Lateral cephalogram CBCT	Impacted	Maxillary anterior region between two permanent incisors	1/2	Both conical	Contiguous with the nasopalatine canal
M	11	Panoramic radiograph Lateral cephalogram CBCT	Impacted	Maxillary anterior region beyond two permanent incisors	2/2	Both conical	Contiguous with the nasopalatine canal and with the above nasal floor

## Data Availability

Data sharing not applicable to this article as no datasets were generated or analyzed during the current study. The present review was not registered.
